# Antimicrobial Activity and Chemical Characterization of a Non-Polar Extract of Saffron Stamens in Food Matrix

**DOI:** 10.3390/foods10040703

**Published:** 2021-03-26

**Authors:** Severino Zara, Giacomo L. Petretto, Alberto Mannu, Giacomo Zara, Marilena Budroni, Ilaria Mannazzu, Chiara Multineddu, Giorgio Pintore, Francesco Fancello

**Affiliations:** 1 Department di Agricultural Sciences, University of Sassari, 07100 Sassari, Italy; szara@uniss.it (S.Z.); gzara@uniss.it (G.Z.); mbudroni@uniss.it (M.B.); imannazzu@uniss.it (I.M.); cmulti@uniss.it (C.M.); 2 Department of Chemistry and Pharmacy, University of Sassari, 07100 Sassari, Italy; albertomannu@gmail.com (A.M.); pintore@uniss.it (G.P.); 3 Mannu Consulting, 291 Milan, Italy

**Keywords:** by-products, liposoluble fraction, antimicrobial activity, polyunsaturated fatty acid

## Abstract

The production of saffron spice generates large quantities of plant by-products: over 90% of the plant material collected is discarded, and a consideration fraction of this waste is plant stamens. This work investigated the chemical composition and the antimicrobial activities of the non-polar fraction extracted from four different saffron flower stamens. The chemical composition of ethereal extracts of the saffron stamens was qualitatively assessed by means of gas–chromatography-mass spectrometry (GC-MS) and nuclear magnetic resonance (NMR) analyses. These analyses revealed ethereal extracts to possess a high polyunsaturated fatty acid content. In vitro antibacterial activity of stamen extracts showed no large differences between Gram-positive and Gram-negative bacteria in terms of minimal inhibitory concentration (MIC). In food matrix microbial analysis of the bacterial strains belonging to the main foodborne pathogen species, including *Staphylococcus aureus* DSM 20231, *Escherichia coli* DSM 30083, and *Listeria monocytogenes* DSM 20600, using low-fat UHT milk, revealed a statistically significant reduction in the number of cells (particularly for *E. coli* and *S. aureus* with a complete elimination of the population of the two target bacteria following incubation in diethyl ether extracts of saffron stamen (DES) at high concentrations tested, both at 37 °C and 6 °C (for 48 h and 7 days, respectively). A synergic effect was observed when the pathogens were incubated at 6 °C with DES. This work shows these by-products to be excellent sources of bioactive compounds, which could be exploited in high-added-value products, such as food, cosmetics, and drugs.

## 1. Introduction

Saffron (*Crocus sativus* Linn.), of the family of Iridaceae, is commonly used for the production of the world’s most expensive spice, used for food flavoring, coloring, and preserving. Saffron spice has long been employed in traditional medicine, and recent studies have demonstrated its medicinal and biological properties [1,2,3,4]. The stigmas, which form a very small percentage of the total flower mass, are collected and processed for spice production. The production of saffron spice generates a number of by-products, with 1 kg of flowers yielding only 15 g of spice. This means that over 90% of the plant material collected ends up being discarded [5,6,7]. Specifically, for each kg of spice, about 63 kg of floral bio-residues (about 53 kg of tepals, 9 kg of stamens, and 1 kg of styles), 1500 kg of leaves, 100 kg of spathes, and 100 kg of corms [1,8] are collected. Therefore, in the frame of the principles of circular economy, the availability of new technological and environmentally friendly solutions to utilize saffron floral waste is an increasing need. New technological and environmentally friendly solutions are thus needed to identify novel applications for saffron floral-waste products in order to engage the principles of circular economy and reduce waste. The valorization of these by-products will also help foster the sustainability of saffron flower cultivation and increase the profitability of this industrial sector by taking advantage from this high-value biomass [1]. Over recent years, many researchers have focused on the biological activity of saffron by-products [1,6,7,9,10,11,12,13,14,15], highlighting their antioxidant, antityrosinase, antidepressant, antinociceptive, and anti-inflammatory activities, their cytotoxic properties against tumor cell lines, their antifungal and antibacterial activities, and their ability to reduce arterial blood pressure [16].

The saffron flower tepals and petals contain considerable amounts of flavonol glucosides, flavonoid glycosides, crocin, and kaempferol, as well as other compounds, such as anthocyanins and lutein diester [15,17,18,19,20], suggesting that these could be good sources of bioactive compounds for the development of functional foods and cosmetic formulations, and a natural color source of anthocyanins for food and biomedical applications [21,22,23].

Saffron stamens are characterized by their high content of ash, protein, soluble sugars [7], and unsaturated fatty acids [24]. On the contrary, the total amounts of polyphenolics, polysaccharides, and flavonoids are lower in the stamens compared with the tepals [16,17].

Of the various analytical techniques that can be employed for the characterization of saffron extracts, nuclear magnetic resonance (NMR) spectroscopy stands out for its ability to generate a metabolic profile in a fast and reliable manner. Methanolic extracts of saffron flower (*Crocus sativus* Linn.) were spectroscopically characterized by Straubinger and co-workers, who reported the presence of glycosidic aroma-related derivatives [25], and by Assimiadis, who employed the proton NMR (^1^H NMR) technique to describe the cis-trans carotenoids [26].

More recently, ^1^H NMR and diffusion-ordered NMR spectroscopy (^1^H DOSY) were used to determine the metabolic profiles of the methanolic extracts of Greek, Spanish, Hungarian, Turkish, and Italian saffron flowers, revealed to be mainly formed of a mixture of linoleic and linolenic acids, phosphatidylcholine, acetic acid, trans and cis crocins, and sugars [27]. In particular, the application of NMR spectroscopy combined with multivariate analysis for the authentication and classification of saffron extracts has emerged as an important new field of research [28].

Few studies addressing the biological activity of saffron stamens have been conducted to date. Menghini et al. [29] found that the stamens exert a protective effect, in the absence of any genotoxic or cytotoxic effects, assessed using in vitro and ex vivo pharmacological models of inflammation and oxidative stress. Furthermore, Montoro et al. found the stamens to exert good antioxidant and antimicrobial activities [10].

Considering the large amount of plant material derived from saffron spice production and the presence of very few reports on the liposoluble fraction of stamens, the aim of the present work was to characterize the chemical composition and biological activity of the diethyl ether extract of the discarded saffron stamens. The diethyl ether extract was characterized by means of gas-chromatography-mass spectrometry (GC-MS) and NMR analyses. Its antimicrobial activity was assessed using the broth dilution method against foodborne pathogens, and evaluated on a food matrix at two different temperatures over time.

## 2. Materials and Methods

### 2.1. Plant Material and Extraction Method

Saffron flower (*Crocus sativum* Linn) stamens were handpicked in Taliouine, Morocco, by a local producer, and from three different areas in Sardinia (Villanova Monteleone, Atzara, and Sardara), Italy. Plant materials were extracted according to Chichiriccò et al. [24] with slight modification. Briefly, saffron stamens were extracted with diethyl ether (sample to solvent ratio 1.5:50 *w*/*v*) at room temperature for 1 h under constant stirring. Then the solvent was removed under vacuum and the collected residue was left at 4 °C until the experiments took place.

### 2.2. NMR Analysis

All manipulations were carried out without using inert gases and solvents, and reagents were employed as received from suppliers. NMR spectra were recorded at 11.7 T using a Bruker NEO 500 spectrometer equipped with a 5 mm pulsed-field z-gradient broad band FO (BBFO) probe and a variable-temperature unit, and were referenced internally to the deuterated solvent. For decoupled ^31^P{^1^H} NMR spectra, 85% H_3_PO_4_ was used as the external standard. Topspin software (4.0.7 version) from Bruker was used for data processing. All the NMR spectra were recorded at 298 K (25 °C) unless otherwise indicated. NMR analyses were conducted using CDCl_3_ on about 10 mg of raw extract as solvent.

### 2.3. GC–MS Fatty Acid Analysis

Fatty acid methyl esters were determined qualitatively according to Angioni and Addis [30]. Briefly, 300 μL of methanol potash were added to a solution of about 10 mg of the raw extract dissolved in 400 µL of diethyl ether, and the mixture was agitated by vortexing for five min. The organic layer was then collected and injected (1 μL) in GC-MS for the analysis.

### 2.4. Antimicrobial Activity

The antimicrobial activities of the different diethyl ether extracts of saffron stamens (from here on referred to as DES) were tested against seven pathogenic bacteria by determining the minimal inhibitory concentration (MIC) using the broth microdilution test. The tested strains were retrieved from DSMZ (Deutsche SammLung von Mikroorganismen und Zellkulturen/the German Collection of Microorganisms and Cell Cultures) and from the culture collection of the UNISS Microbial Collection (University of Sassari), Italy. The strains and the culture conditions utilized are indicated in Table 1.

First, a sterile water stock solution of 1% dimethyl sulfoxide(DMSO), 5% Tween 80, and a concentration of 36 mg/mL DES was prepared. Serial two-fold dilutions were then made to give a final concentration range from 0.14 mg/mL to 36 mg/mL. Then, 100 μL of the double-strength solutions were dispensed into 96-well micro-dilution plates. The growth method was used for inoculum preparation, according to the National Committee for Clinical Laboratory Standards (NCCLS) Approved Standard M07-A9 [31]. The adjusted inoculum suspension was diluted in 2× cation adjusted Muller Hinton Broth (MHB), and 100 μL aliquots were added to each well in the 96-well micro-dilution plate already containing 100 μL of double strength DES dilutions (Oxoid, Basingstoke, England). After inoculation, each well contained approximately 5 × 10^5^ CFU/mL. The plates were then incubated at 37 °C for 24 h. After incubation, minimal inhibitory concentrations (MICs) (μL/mL) values were determined as the lowest ethereal extract concentration that inhibited visible growth of the tested microorganism, indicated by the absence of turbidity. Each tray included a growth control well (cation-adjusted MHB without DES), a sterile (uninoculated) well, and a negative control well (MHB medium with 1% DMSO and 5% Tween 80). Each tray was replicated in quadruplicate (four technical replicates for each microbial strain tested) and the experiments were repeated twice.

The minimum bactericidal concentration (MBC) was also determined. Ten microliters of samples from wells where no growth was detected were plated onto Brain Heart Infusion agar (BHI) (Oxoid, Basingstoke, UK) and incubated at 37 °C for 24 h. At the end of the incubation period, the lowest concentration with no growth (no colony) was defined as the MBC.

### 2.5. Evaluation of Antimicrobial Activity in a Food Matrix

#### 2.5.1. Preparation of milk spiked with DES Extract

Low-fat UHT milk was used as the food matrix in order to test the antimicrobial activity of four different DES extracts. The DES extracts were resuspended in low-fat UHT milk containing 2% Tween 80 with a final concentration ranging between 9 mg/mL and 2.5 mg/mL. Two positive controls were prepared using low-fat UHT milk alone and low-fat UHT milk supplemented with 2% Tween 80.

#### 2.5.2. Preparation of Bacteria Inoculum

Three of the seven strains previously analyzed were used for this test. Specifically, *Staphylococcus aureus* DSM 20238, *Escherichia coli* DSM 30083, and *Listeria monocytogenes* DSM 20600 were used as target bacterial strains. These species were grown overnight in BHI broth (WVR, Milano, Italy) at 37 °C. Afterwards, 1 mL of culture was centrifuged at 14,000× *g* for 3 min; after discarding the supernatant, the pellets were washed three times with 0.89% NaCl solution, and resuspended in the same solution. The cellular suspensions were used to inoculate the milk spiked with every DES extract. The cellular concentration of the suspension was determined by means of the plate count method using BHI agar (WVR, Milano, Italy).

#### 2.5.3. Inoculation and Growth Condition

Low-fat milk, prepared as described above, was inoculated with the cellular suspension of the three different above-cited species to obtain a final concentration of around 6 Log_10_ CFU/mL. For each concentration and temperature tested, a series of three polypropylene sterile tubes containing 5 mL low-fat milk was prepared. After inoculation, the tubes were incubated at 37 °C and 6 °C for 48 h and 7 days, respectively. The growth dynamic of bacterial strains was determined using the colony count methods at 24 and 48 h for the plates incubated at 37 °C, and at 48 h and 7 days for the plates incubated at 6 °C.

### 2.6. Statistical Analysis

Microbial counts were log-transformed to obtain a normal distribution. Data were analyzed by analysis of variance (ANOVA) using the concentration of DES and time as fixed factors. When a significant effect was observed (*p* < 0.05), the differences between means were separated using the Tukey-Kramer multiple comparisons test. SPSS software, version 19.0, was used to carry out the statistical analyses.

## 3. Results and Discussion

### 3.1. NMR Spectroscopy and GC Methyl Fatty Acids Esters Analysis

**Figure 1:**
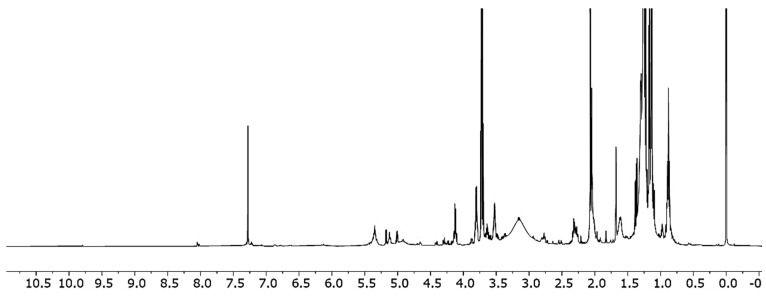
^1^H nuclear magnetic resonance (NMR) of diethyl ether extracts of saffron stamens (CDCl_3_).

**Figure 2:**
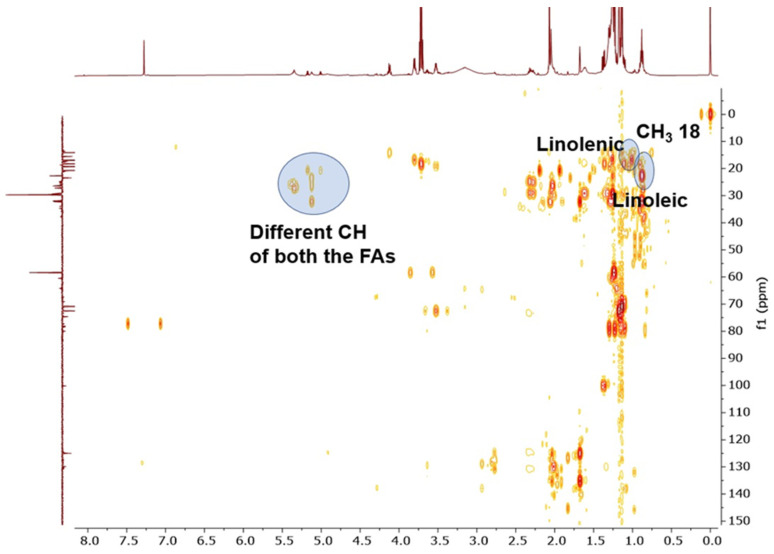
Heteronuclear single quantum correlation (HSQC) analysis of diethyl ether extracts of saffron stamens.

The raw diethyl ether extract of saffron stamens was subjected to qualitative chemical analysis by NMR spectroscopy: ethereal extracts were examined by ^1^H, ^31^P, HSQC, and ^13^C DEPT experiments. By referring to the detailed characterization of NMR signals relative to the main metabolites present in polar saffron extracts published by Sobolev and co-workers [26], we were able to confirm the presence of linoleic and linolenic fatty acids represented in the ^1^H NMR by the signals for -CH_3_ at 0.95 ppm, -CH_2_- at 1.6 ppm, and the double bond at 5.35 ppm. A typical crocetin fingerprint was observed at 2.01, 2.33, and 2.7 ppm (Figure 1). This attribution by ^1^H NMR was confirmed by both HSQC and ^13^C DEPT analyses (Figure 2).

**Figure 3:**
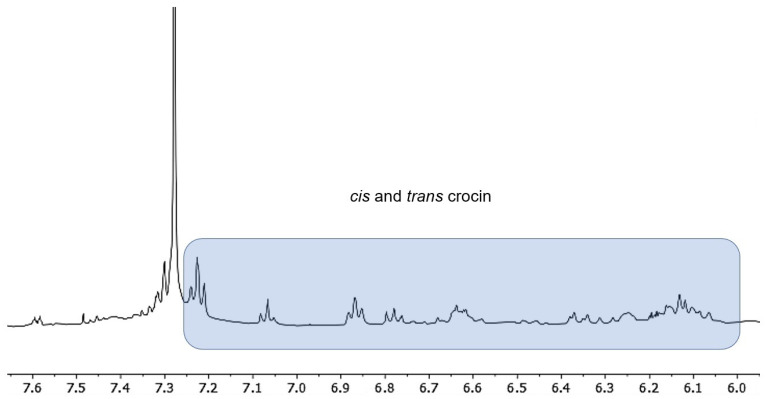
Portion of the ^1^H NMR spectrum indicating the presence of crocin.

A more detailed analysis of the ^1^H NMR spectrum revealed the presence of a mixture of cis and trans crocin present in traces in the ethereal extract (but yet visible at NMR) due to its low solubility in non-apolar solvents such as diethyl ether (Figure 3).

^31^P NMR excluded the presence of any phosphorous derivatives. In addition to linoleic and linolenic fatty acids, already detected by NMR analysis, GC-MS analysis of the methyl esters of the fatty acid, obtained by reacting the diethyl ether extract with a mixture of KOH and methanol, revealed the presence of lauric, myristic, palmitic, vaccenic, and oleic acids (data not reported). These results lie in agreement with those reported by Chichiriccò et al. [24,32], who found linoleic, linolenic, and palmitic acid to be the predominant fatty acids in saffron stamens.

### 3.2. Antimicrobial Activities In Vitro and in Food Matrix

No differences were found in the MICs values obtained for the different bacteria strains tested or between the four sources of saffron stamen (Table 2).

No differences were detected between Gram-positive and Gram-negative bacteria. The antimicrobial activity of stamens and other flower bioproducts can be ascribed to their components; in particular, to flavonoid [33], polyphenols [34,35], terpenes [36], fatty acid, and polyunsaturated fatty acids, such as omega acids (linolenic acid) [32,37]. Several studies showed that polyunsaturated fatty acid (PUFA), in particular those belonging to omega-3 and omega-6 polyunsaturated fatty acids series such as linolenic acid (LNA) and its derivatives (eicosapentaenoic acid (EPA) and docosahexaenoic acid DHA) have an antimicrobial activity. This activity is linked to the ability of fatty acid to alter cell membranes, causing the disruption of cell-to-cell communication, adenosine triphosphate (ATP) production, an alteration in membrane hydrophobicity and fatty acids (FA) synthesis, cellular leakages via increasing membrane poles, and disruption of the electron transport system (reviewed by [38]). Here, the most sensitive strain was *S. aureus* (MIC, 4.5 mg/mL). Similarly, Gandomi Nasrabadi et al. [39] showed that aqueous, ethanolic, and methanolic extracts of saffron petals exerted more antimicrobial activity against *S. aureus* than *E. coli*. It has been reported that Gram-positive bacteria are usually more sensitive to plant-origin antimicrobials compared with Gram-negative bacteria, which are usually more resistant. The resistance of the Gram-negative bacteria has been attributed to their cell wall structures, in particular their outer membranes, which may resist the penetration of the active compounds from plant extracts [40,41]. For the same reason, the Gram-negative bacteria analyzed herein showed the highest MBC, whereas no differences were observed in *L. monocytogenes* DSM 20600 in the values of MIC and MBC. By contrast, dos Santos et al. [42] found intra-species variability in *S. aureus* in the action of diethyl ether extract of *Indigofera suffruticosa*, as also demonstrated in other works using different plant extracts [43,44,45]. Elansary et al. [46] reported the weak antibacterial activity of diethyl ether extract of *Ceratostigina plumbaginoides*, expressed as MIC and MBC in the range of 0.07–0.28 and 0.14–0.51 mg/mL, respectively, which were lower than the values of MIC and MBC found in this work. Jastaniah [47] reported that the most active extract from *Crocus sativus* flowers was the methanol extract, with MIC values ranging from 50–75 µg/mL, depending on the bacterial species tested. Compared with other types of extract, such as methanol extract, ethanol extract, water, and acetone extract, several authors report diethyl ether extracts to present the lowest levels of antimicrobial activity [46,48]. Comparing the DES with the *Foeniculum vulgare* diethyl extracts, the latter showed higher antibacterial activity than the former [49]. Similar results were also found with *Xeranthemum* species [50]. Mir et al. [36] observed higher antibacterial activities for petal, style, and leaf extracts with respect to the stamen, whereas the stamen extracts showed higher antifungal activities. Kakouri et al. [22] observed that the minimum amount of saffron tepal extract to exhibit antimicrobial activity was 1.5 mg, whereas just 5 mg of the same extract were sufficient to successfully inhibit all microorganisms tested. Asgarpanah et al. [51] reported that the MICs and MBCs of various extracts of petals and stamens were higher than the MICs found in this work. The same authors found that the ethyl acetate (a non-polar solvent) sub-fraction of *C. sativus* stamens showed the best antibacterial activity versus *Shigella dysenteriae* PTCC 1188, *Salmonella thiphi* ATCC 19430, and *E. coli* ATCC 25922.

Mir et al. [36] found the petroleum ether extract of saffron stamen inhibited the growth of *Proteus mirabilis* (MTCC-425), *Malassezia furfur* (MTTC-1374), and *Trichophyton rubrum* (MTCC-7859).

**Figure 4:**
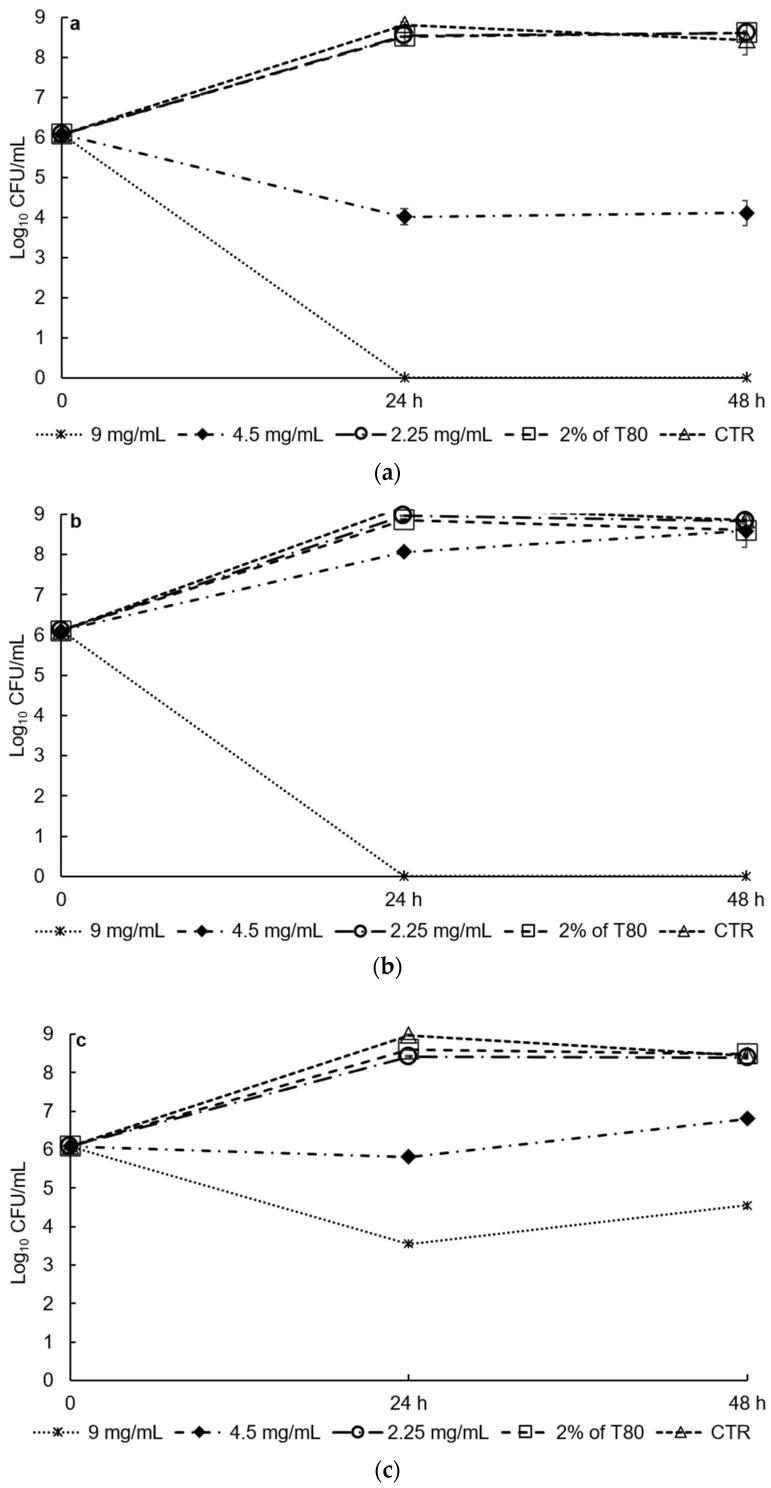
Effects of different concentrations of DES (9, 4.5, and 2.5 mg/mL) on the growth of (**a**) *S. aureus* DSM 20231, (**b**) *E. coli* DSM 30083, and (**c**) *L. moncytogenes* DSM 20600; low-fat UHT milk was used as food matrix, and antibacterial activity of the extracts was tested at 37 °C for 48 h by comparing bacterial growth with control conditions (low-fat UHT milk alone, “CTR”; and low-fat UHT milk supplemented with 2% of Tween 80, “2% of T80”). Values are the average of four biological replicates (see materials and methods).

**Figure 5:**
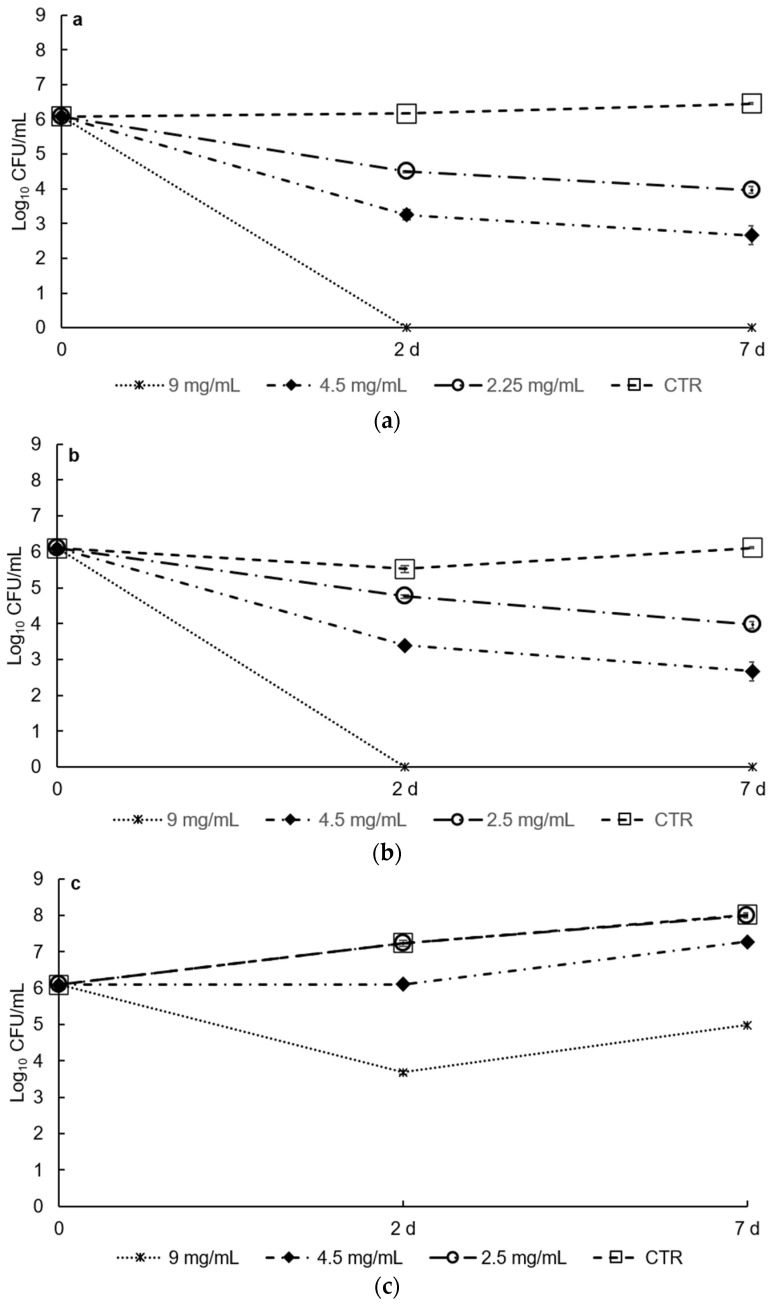
Effects of different concentrations of DES (9, 4.5, and 2.5 mg/mL) on the growth of (**a**) *S. aureus* DSM 20231, (**b**) *E. coli* DSM 30083, and (**c**) *L. moncytogenes* DSM 20600; low-fat UHT milk was used as food matrix, and antibacterial activity of the extracts was tested at 6 °C for 7 days by comparing bacterial growth with control conditions (CTR, low-fat UHT milk alone). Values are the average of four biological replicates (see materials and methods).

DES extracts showed good antibacterial activity against *S. aureus* DSM 20231 and *E. coli* DSM 30083 inoculated into low-fat UHT milk when used at the highest concentration tested (9 mg/mL) at both 37 °C and 6 °C (Figure 4 and Figure 5).

Indeed, the initial number of cells (6.1 log_10_ CFU/mL) of both species markedly decreased after 48 h of incubation. On the contrary, the cell number of *L. monocytogenes* incubated at 37 °C was significantly reduced (*p* < 0.001) in the first 24 h (from 6.1 log_10_ CFU/mL to 3.6 log CFU/mL), but then increased, reaching 4.6 log_10_ CFU/mL at 48 h. The highest concentration used (9 mg/mL) corresponded to the minimal bactericidal concentration (MBC) for *S. aureus* and *L. monocytogenes*, and the minimal inhibition concentration (MIC) for *E. coli*. The results obtained in this work contrast with those frequently reported for food matrices. In particular, the DES extracts showed better antimicrobial activity against *E. coli* in low-fat milk compared with in vitro.

The food matrix bioactivities revealed in this work (using low-fat UHT milk as medium) are of considerable interest because plant-derived compounds usually degrade when applied to food systems, usually due to their volatile nature [52,53] and owing to their effective concentrations that are reduced as a result of interactions with surrounding lipophilic food components, such as proteins and fats [52,54,55], and also because these compounds have limited solubility in the aqueous phase [52,54,55].

Finally, the antimicrobial synergic effect between DES extracts and a low temperature was studied. Mild hurdle technologies, which involve multiple simultaneous preservation approaches, are currently applied in food storage and preservation strategies to reduce the use of chemical additives and to increase their environmental and economic sustainability [46]. DES extracts combined with low temperature (6 °C) showed antimicrobial activity against *S. aureus* and *E. coli* even at the lowest concentration investigated (2.25 mg/mL). At this concentration, a reduction of about 1.5 logarithmic CFUs was observed compared with the initial value (6.1 log_10_ CFU/mL) after two days for both microorganisms tested (Figure 5, panels a and b) (*p* < 0.001). At a DES concentration of 4.5 mg/mL, the reduction was even more pronounced with a significant decrease (*p* < 0.001) in a CPU of 3 log units. On the contrary, the effect of temperature on *L. monocytogenes* was less evident, which may be due to the ability of this species to resist, and grow at, low temperatures, although a significant reduction was observed in the initial cell number when the higher concentration of DES extract was used (*p* < 0.001).

## 4. Conclusions

In this work, we show that the liposoluble fraction of saffron flower stamens—a part of the plant discarded during the production of saffron spice—is rich in linoleic, linolenic, and palmitic fatty acids, and exhibits strong antimicrobial activity, both in in vitro and in the food matrix, against some strains of the most common food-borne pathogenic bacterial species, including *S. aureus* and *E. coli*. These activities did not differ for extracts obtained from saffron flowers sourced from different locations (Morocco and Sardinia). We also discovered that the antimicrobial activities tested in the food matrix, using low-fat UHT milk as food matrix, persisted over the time; test tubes inoculated with *S. aureus* and *E. coli* in milk plus DES continued to be free of bacterial colonies after one year (unpublished results). The results obtained in this work will help maximize the exploitation of saffron flower harvest residues as important sources of bioactive compounds to be used in the medical or food industry.

## Figures and Tables

**Table 1:** List of microorganisms, medium, and culture conditions used in this work for testing the antimicrobial activity of diethyl ether extracts of saffron stamens (DES).

Bacteria	Source	Medium	Temperature and Time of Incubation
*Staphylococcus aureus* DSM 20231	DSMZ	BHI	37 °C × 24 h
*Listeria monocytogenes* B	UNISS	BHI	37 °C × 24 h
*Listeria monocytogenes* E	UNISS	BHI	37 °C × 24 h
*Listeria monocytogenes* C	UNISS	BHI	37 °C × 24 h
*Listeria monocytogenes* DSM 20600	DSMZ	BHI	37 °C × 24 h
*Salmonella enterica subsp. bongori* DSM 13772	DSMZ	BHI	37 °C × 24 h
*Escherichia coli* DSM 30083	DSMZ	BHI	37 °C × 24 h

DSMZ, Deutsche SammLung von Mikroorganismen und Zellkulturen/German Collection of Microorganism of Cell Cultures; UNISS Microbial Collection (University of Sassari), Italy.

**Table 2:** Minimum inhibitory concentrations (MIC, mg/mL) and minimum bactericidal concentrations (MBC, mg/mL) of the diethyl ether extracts of saffron stamens against the microorganisms tested.

Bacteria	MIC *	MBC *
*Staphylococcus aureus* DSM 20231	4.5	9
*Listeria monocytogenes* B	9	9
*Listeria monocytogenes* E	9	9
*Listeria monocytogenes* C	9	9
*Listeria monocytogenes* DSM 20600	9	9
*Salmonella enterica* subsp. *bongori* DSM 13772	9	18
*Escherichia coli* DSM 30083	9	18

* Values for extracts obtained from different saffron stamens sources did not vary for the bacteria species tested.
